# Co-infection with *Strongyloides stercoralis* hyperinfection syndrome and *Klebsiella* in a nephrotic syndrome patient: A case report

**DOI:** 10.1097/MD.0000000000018247

**Published:** 2019-12-10

**Authors:** Wei-Li Wang, Qi-Wu Zhang, Sha Tang, Feng Chen, Jing-Bo Zhang

**Affiliations:** Department of Nephrology, the Second Affiliated Hospital (Xinqiao Hospital), Army Military Medical University (Third Military Medical University), Chongqing, China.

**Keywords:** hyperinfection syndrome, immunosuppressed patients, nephrotic syndrome, *Strongyloides stercoralis*

## Abstract

**Rationale:**

Patients with chronic *Strongyloides stercoralis* infection are usually asymptomatic; therefore, their condition is easily overlooked. In immunosuppressed patients, mortality is high because of disseminated infection and hyperinfection. This report describes a fatal *S stercoralis* hyperinfection in a patient with nephrotic syndrome after treatment with steroids.

**Patient concerns:**

A 70-year-old male presented with a history of progressive edema, skin infection, persistent fever, cough, intermittent abdominal pain, and progressive respiratory failure after steroid treatment.

**Diagnosis:**

Nephrotic syndrome; cellulitis; *S stercoralis* hyperinfection; Klebsiella pneumonia.

**Interventions:**

During the first hospital admission, the patient was administered full-dose glucocorticoid and antibiotic therapy after suffering from cellulitis. During the second admission, he was diagnosed and treated for normal digestive discomfort and a bacterial infection. The patient had progressive respiratory failure and was placed on a ventilator. He was immediately treated with albendazole when *S stercoralis* was found in samples of his sputum and feces.

**Outcomes:**

The patient died despite treatment with albendazole and antibiotic therapy.

**Lessons:**

It is essential to consider the possibility of *S stercoralis* infection in immunosuppressed patients with nephrotic syndrome. Given the lack of classic manifestations and high mortality rate of advanced disease, continuous monitoring, early diagnosis, and proper treatment are imperative.

## 1 Introduction

*Strongyloides stercoralis* is an intestinal parasite that spawns larvae in the soil and mainly infects humans. Most cases of *S stercoralis* are distributed in tropical, subtropical, and temperate regions.^[1]^ From 1973 to 2013, 330 cases were reported in China, mainly in the southern regions.^[2]^ Those chronically infected with *S stercoralis* are usually asymptomatic and are easily overlooked by healthcare workers. The immunosuppressed population is more vulnerable to disseminated infection and is more likely to develop hyperinfection. Many studies on *S stercoralis* have focused on organ transplant recipients and patients with malignant tumors, since these individuals often receive multi-target immunosuppression treatment and therefore have severe immunodeficiency. We reviewed the literature and report a case of a fatal *S stercoralis* hyperinfection in a patient with nephrotic syndrome.

## 2 Case report

A 70-year-old male suffered progressive generalized edema after consuming stale crabs, with only mild abdominal discomfort and no fever or other symptoms. Before this, he was healthy and did not have a history of digestive diseases, diabetes, or chronic obstructive pulmonary disease. The man was a native of Chongqing, the subtropical area in southwest China. He used to be a soldier; he fought in the Vietnam War and joined the police force after returning to his hometown.

In the hospital, his initial vitals were BP 108/78 mmHg, HR 111, respiratory rate 22, and oxygen saturation 98%. Initial laboratories included white blood cells 13.32 × 10^9^/L (neutrophils% 77.5%; lymphocytes% 13.31%; eosinophils% 0.5%), normal hemoglobin and platelets, albumin (ALB) 14.5 g/L, globulin (GLB) 19.9 g/L, alanine aminotransferase (ALT) 78.7 IU/L, aspartate aminotransferase (AST) 90.9 IU/L, creatinine (Cr) 134 μmol/L, 24-hour urine protein 9.61 g, and negative antinuclear antibody spectrum (ANAs) and anti-neutrophil cytoplasmic antibodies (ANCA). Chest X-ray showed mild emphysema but no sign of infection. The patient was diagnosed with nephrotic syndrome but was unable to undergo pathological biopsy due to a renal cyst. He was administered full-dose glucocorticoid therapy alone, with no other immunosuppression. Three weeks later, while still under this treatment, the patient suffered lower limb cellulitis. His procalcitonin (PCT) was 0.3 ng/ml, and he was administrated mupirocin ointment and IV cefuroxime. After those treatments, his status improved and he continued to take oral glucocorticoids after discharge from the hospital.

**Figure 1:**
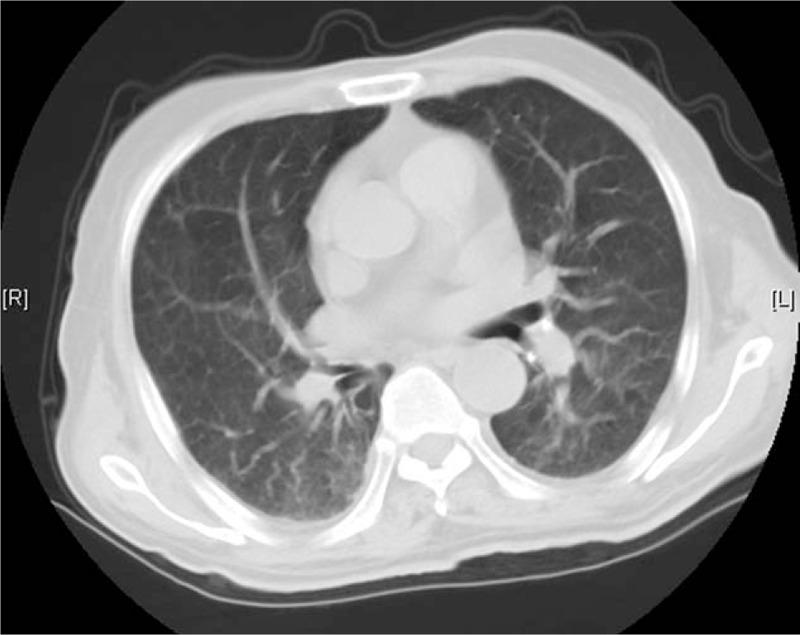
A chest computed tomography showing interstitial pneumonia.

**Figure 2:**
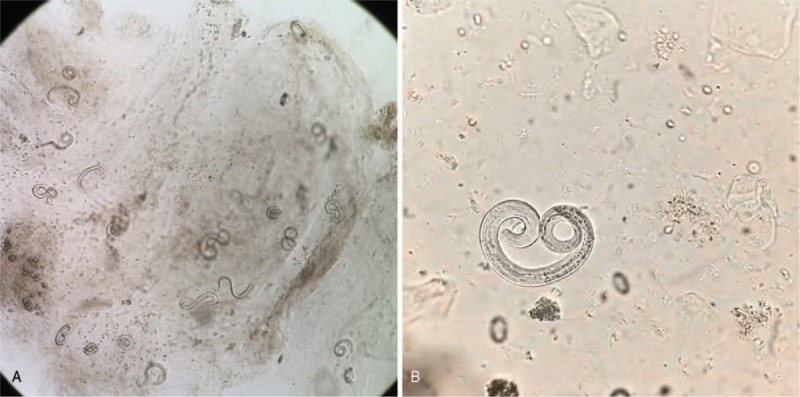
A large number of *Strongyloides stercoralis* are found in the sputum (A), and are further identified as filariform larvae (B).

**Figure 3:**
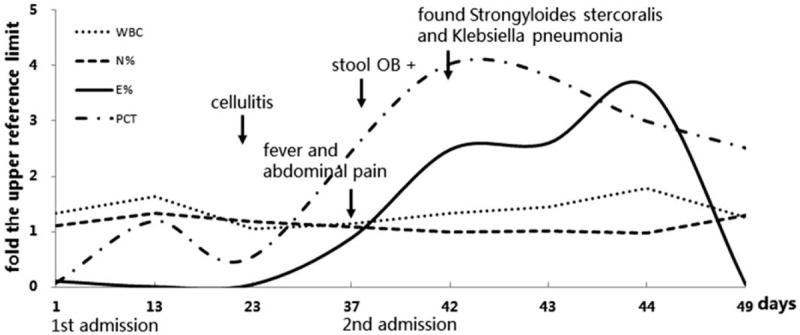
The course of the disease. Lines showed as fold the upper reference limit indicate WBC absolute count, neutrophil %, eosinophil % and procalcitonin, respectively. WBC, white blood cell.

However, over the next ten days, the patient seemed to get worse and had to return to the hospital due to persistent fever, cough, and intermittent abdominal pain. Initial vitals on admission were temperature 37.8 °C, BP 90/60 mmHg, HR 125, respiratory rate 26, and oxygen saturation 95%. Laboratory tests showed white blood cells 12.36 × 10^9^/L (neutrophils %: 83.5%; eosinophils %: 0.7%). Sputum smear and culture were negative. Imaging examinations included CT scans of the chest, which reported interstitial pneumonia (Fig. 1), and the abdomen, which reported no specific findings. The patient was diagnosed with normal gastrointestinal discomfort and pulmonary bacterial infection. A proton pump inhibitor, cefoperazone sodium, and sulbactam sodium were administered. However, the patient then started display hemoptysis, passed occult blood-positive stool, and gradually fell into a state of hyperpyrexia and drowsiness. Soon, *Klebsiella pneumoniae* (which was sensitized to the previous antibiotic), and unexpectedly, a large number of *S. stercoralis* larvae (Fig. 2) were found in repeated sputum specimens. Meanwhile, the parasite was also found in a repeated brown stool specimen. Blood samples showed eosinophils% reached 18.1% and PCT was 1.01 ng/ml. Because ivermectin is not available in our region, albendazole (400 mg/day) was administered for 3 consecutive days but there was no progress. Subsequently, a mechanical ventilator was administered due to continued severe hypoxia and respiratory failure. The patient was unable to maintain his blood pressure and eventually died after co-infection with *S stercoralis* hyperinfection and the *K pneumoniae* found 1 week later. The course of this case has been summarized in Figure 3. The authorized relative of patient has provided informed consent for publication of the case.

## 3 Discussion

Infection with *S stercoralis* is often initiated through contact with soil contaminated with infectious larvae that penetrate the skin and migrate via the circulatory system to the lung, where they are expectorated and travel through the trachea to be swallowed back down to the gastrointestinal tract.^[3,4]^ This migration is usually clinically asymptomatic and therefore often goes unnoticed by the host. After migration, the larvae mature into adult roundworms and release eggs in the gastrointestinal tract. Some eggs are excreted and some transform into infectious larvae and re-infect the host in a process called autoinfection. An infected patient may survive without any symptoms for decades if his or her immune system is not compromised. However, a patient who has received steroid therapy is more susceptible to fatal dissemination and hyperinfection.^[5,19]^

The innate and adaptive immune system, both profoundly modulated by strongyloidiasis, are vital to the prevention of *S stercoralis* disseminated infection. Larval migration and proliferation are facilitated due to varying degrees of inhibition in an immunosuppressed host. As part of the innate immune system, eosinophils play a prominent role in parasitic infections and have been confirmed to kill worms and act as antigen-presenting cells (APCs) for the initiation of Th2 immune responses in strongyloidiasis.^[20]^ Recently findings proved that neutrophil and mast cell activation may play another important role in defending against *S stercoralis* infection.^[21]^ Studies on adaptive immunity in strongyloidiasis have reported that the CD4+ T cell subsets Th1, Th2 and Th17 showed susceptibility or resistance to infection.^[22]^

Further migration of larvae causes noticeable symptoms, mainly in gastrointestinal tract and lung, including emesis, diarrhea, abdominal pain, wheezing, and hemoptysis.^[4]^ Some patients diagnosed with strongyloidiasis can develop hyperinfection syndrome, wherein larvae proliferation spirals out of control, resulting in disseminated larval infection. This result is especially common if the patient has received immunosuppressants such as steroids and chemotherapeutics or has become immunocompromised by virtue of chronic strongyloidiasis. Hyperinfection can cause multiple organ failure and death. The mortality rate of *S stercoralis* hyperinfection ranges from 87% to 100%.^[5,6]^ In this case, our patient was administered prednisone. He subsequently suffered a persistent fever, hemoptysis, and abdominal pain. The chest CT showed interstitial pneumonia and both *S. stercoralis* and *K pneumoniae* were found in the sputum. In the end, he developed severe hypoxia and respiratory failure. It is easy to ignore and difficult to diagnose strongyloidiasis before finding evidence of parasitic pathogens. Many patients are either asymptomatic or exhibit non-specific symptoms that are indistinguishable from those of other diseases. Although eosinophilic infiltration is associated with parasitic infections and the ratio of eosinophils increased dramatically when our patient was confirmed to be infected with *S stercoralis*, this indicator is highly variable and is not a reliable tool for prediction or diagnosis.^[7]^ Serological IgG testing of larval antigens has a sensitivity of approximately 80% and a specificity of 97%,^[8]^ but it is less often used in clinical practice. Therefore, parasite detection remains the gold standard for diagnosis. In the treatment of strongyloidiasis, ivermectin 200 μg/kg orally daily for 2 weeks is recommended, as it is well tolerated with few side effects.^[7,9]^ Because ivermectin was absent in our region, albendazole was a reasonable alternative. Nevertheless, cure rates for albendazole are lower than ivermectin,^[10]^ therefore ivermectin should be administered as a first-line treatment whenever possible. Furthermore, Henriquez-Camacho et al showed that although the parasitological cure of ivermectin and albendazole is similar, ivermectin results in more people cured than albendazole.^[9]^

In the 8 reported cases of nephrotic syndrome associated with strongyloidiasis (Table 1), the initial presentation was related to the features of nephrotic syndrome. In most patients, clinical features of strongyloidiasis manifested within 1 or 2 months of receiving steroid treatment. Unlike patients who must undergo multi-target immunosuppression after a kidney transplant^[11]^ or for malignant tumors,^[12]^ steroid alone was the main treatment for these eight patients. Eosinophils were high in 6 of 8 cases, and 3 cases had a concomitant bacterial infection. In our case, the patient suffered cellulitis during treatment, which made him more susceptible to infection after immunosuppressive therapy. In addition to his immunosuppression, the patient's travel to Vietnam also put him at high risk of *S stercoralis* hyperinfection.

**Table 1:**
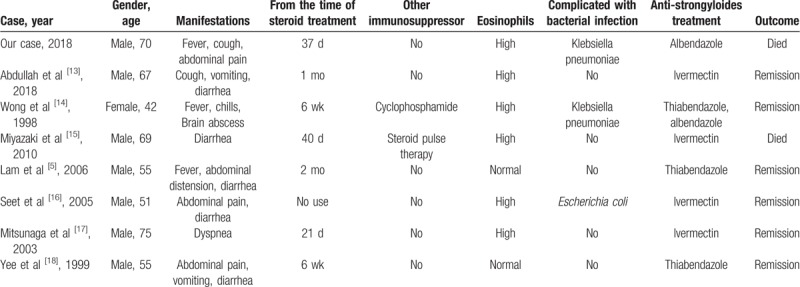
Clinical characteristics of patients in eight reported cases of nephrotic syndrome associated with strongyloidiasis.

Due to the non-specific nature of strongyloidiasis symptoms, of strongyloidiasis it is easy to neglect parasitic infection and therefore delay screening and treatment. This is especially true if there is an accompanying bacterial infection. Hence, ongoing monitoring of any new or progressive symptoms, eosinophil ratio, and repeated examination samples will contribute to timely diagnosis and treatment of strongyloidiasis and will decrease the risk of hyperinfection. Finally, re-establishing immune homeostasis may play a crucial part in the survival of immunosuppressed patients.

## Acknowledgments

We thank Dr. Peng Tang for providing photos and videos of clinical samples.

## Author contributions

**Investigation:** Qiwu Zhang, Feng Chen.

**Project administration:** weili wang, Jingbo Zhang.

**Resources:** Feng Chen.

**Supervision:** Sha Tang.

**Writing – original draft:** weili wang.

**Writing – review & editing:** weili wang, Qiwu Zhang, Sha Tang, Jingbo Zhang.

weili wang orcid: 0000-0002-3502-2360.
